# Supplementary material for: The collective burst mechanism of angular jumps in liquid water
Source: Nat Commun. 2023 Mar 11;14:1345. doi: 10.1038/s41467-023-37069-9 (PMC10008639; doi:10.1038/s41467-023-37069-9)
Supplement: Supplementary file 1 — Supplementary Information [file 41467_2023_37069_MOESM1_ESM.pdf]

Supplementary Information

The Collective Burst Mechanism of Angular Jumps in Liquid Water

Adu Offei-Danso[1,2], Uriel N. Morzan[1], Alex Rodriguez[1,3], Ali Hassanali[1], Asja Jelic[1]

1 Condensed Matter and Statistical Physics, The Abdus Salam International Centre for Theoretical Physics, 34151 Trieste, Italy
2 International School for Advanced Studies (SISSA), 34136 Trieste, Italy
3 Dipartimento di Matematica e Geoscienze, Università degli Studi di Trieste, via Alfonso Valerio 12/1, 34127, Trieste, Italy

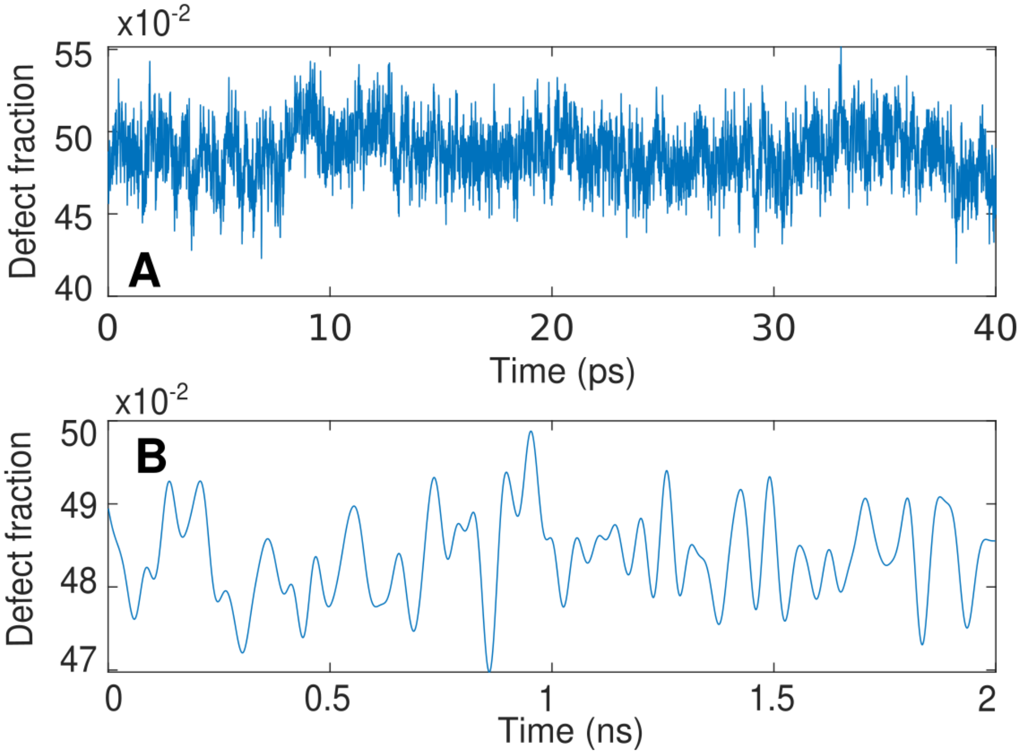

Supplementary Figure 1. Fluctuations in the local topology of the water hydrogen bond network. (A) Unfiltered times series of the fraction of defects (water molecules with non-tetrahedral local topology) on short time scales shows large defect oscillations, reflecting processes in the network that lead to the creation and annihilation of up to 10-20 defective water molecules in the network. (B) Time series of the fraction of defects over a total period of 2 ns of the MD simulation.

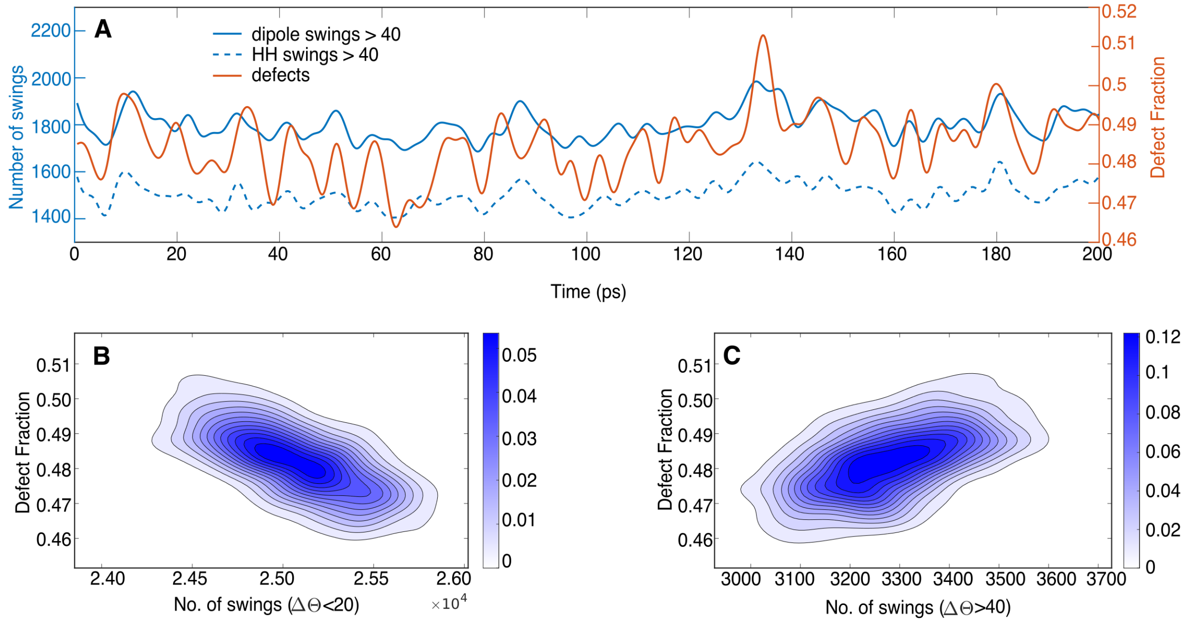

Supplementary Figure 2. Correlation between the number of simultaneous large angular swings and the fluctuations in the local topology of the water H-bond network for SPC/E water model of 1019 molecules. This is similar to Fig.3 in the main text, except that here we reduce the threshold for the minimal magnitude of what is considered a large swing from 60° to 40°, therefore including more angular swings. (A) Time series of the number of molecules in the H-bond network performing large angular swings (amplitude larger than 40°) at each moment of time as detected from the observation of the dipole vector (blue full line) and HH vector (blue dashed line). At each moment of time, we count the number of swings happening in the system within a time window of 1ps around it. We superimpose these time series with the time series of the fraction of molecules in the H-bond network that are defective, i.e. with non-tetrahedral local topology (red). As in the case of swings with $\Delta\Theta > 60$, also here we observe fluctuations of the order of tens of picoseconds in all three curves that often appear to be correlated in time, even though the peaks of the number. (B) Density plot of the fraction of defects in the H-bond network with respect to the number of molecules in the network performing small angular swings ($\Delta\Theta < 20$) within 1ps. Anti-correlation between these two quantities means that when there are more molecules with defective local topology, the less small-amplitude angular swings occur in the H-bond network. We find the correlation coefficient to be -0.7390 ± 0.0089, with $p < 0.01$. (C) Density plot of the fraction of defects in the H-bond network with respect to the number of large-amplitude angular swings ($\Delta\Theta > 40^\circ$) within 1 ps. Correlation between these two quantities indicates that the more the local topology in the H-bond network is defective, the larger is the number of molecules that perform large-amplitude angular swings. The correlation coefficient found is 0.57 ± 0.02, with $p < 0.01$

Supplementary Note 1

In Supplementary Fig.3, we looked at the water molecules that concurrently perform large-amplitude swings and examined how much correlated are the parts of their angular trajectories around the times of these large swings. In particular, we calculated the Pearson coefficients between the HH and DP vector time series of different molecules over a certain time interval of length dT that encompasses the detected large angular swings. In order to do that, for every molecule, we selected the time series of the component of the HH or DP vector with the largest change in magnitude during that time interval dT. In that way, we approximated the angular motion of each water molecule by looking at the direction with the most significant change within the time interval of consideration. We found that an average duration of a large-amplitude angular swing is around 0.1 ps (see Fig. 4a and Supplementary Fig. 4a), therefore, we take dT to be 0.1 ps or longer.

For varying time interval widths dT, ranging from 0.1 ps to 100 ps, we extracted the Pearson correlation coefficients between molecules undergoing large angular swings greater than 60°, as described above. We then constructed probability density estimates from the Pearson correlation coefficients. On average, 50 out of 1019 waters are found to undergo large angular fluctuations within 0.1ps. Therefore, for these results to be statistically significant, we considered different time intervals of length dT in the trajectory, by shifting the initial time of the window of consideration by dT, in order to avoid overlapping statistics. Each probability density estimate was constructed using 10,000 Pearson coefficients. Finally, the probability distributions for molecules undergoing large angular swings were then contrasted with distributions generated by computing the Pearson correlation coefficients between all water molecules over time interval dT in a similar manner. The results of this analysis are shown in Supplementary Fig.3.

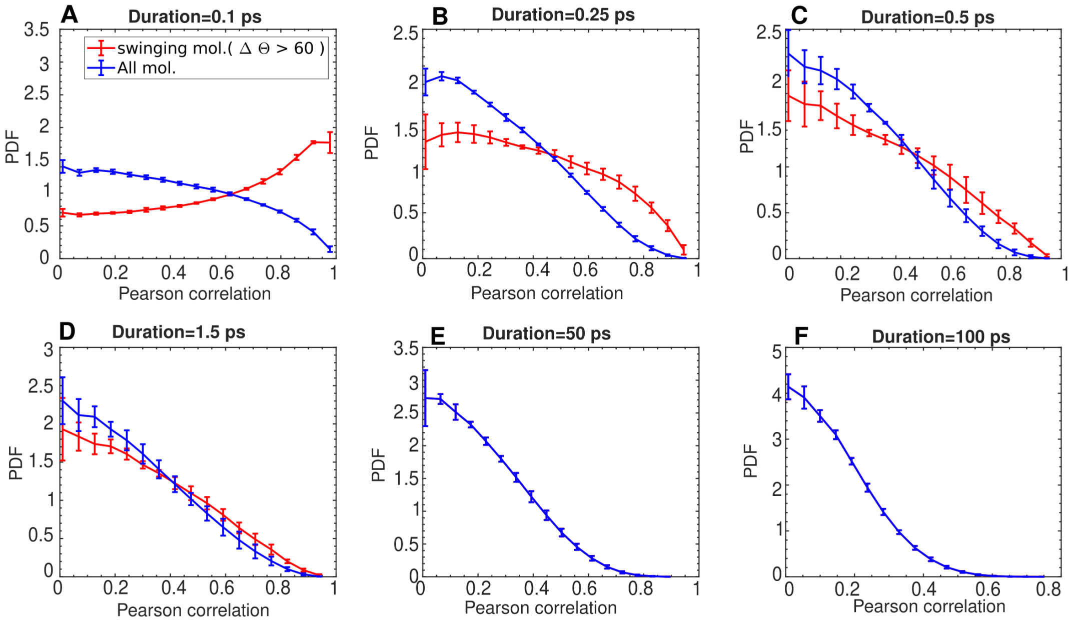

Supplementary Figure 3. Correlation in time of the angular trajectories of water molecules concurrently performing large swings ($\Delta\Theta > 60°$). (A,B,C) In the top three panels, we plot the probability distribution functions of the Pearson correlation coefficient between the HH vector trajectories of length 0.10, 0.25, and 0.50 ps encompassing the angular swings. We show the difference in the correlations between the time series of the molecules with large-amplitude swings (red), with respect to those of all molecules that perform angular swings (blue) within the time window of interest. While for the shortest time interval the correlation between the large swings (red curves) is typically high (peak of the curve is close to 1), as the time interval increases, the time series become more and more uncorrelated and we find that the two distributions almost overlap for 0.5 ps. On the contrary, when we look at the same length trajectories for any other two molecules, they are typically much less correlated (blue curves) even for short times, as expected since motion of any two water molecules in the system is not expected to be correlated. (D,E,F) Bottom panels show the distributions of the Pearson correlation coefficient for the parts of the trajectories of length 1.5, 50, and 100 ps, respectively. While the distributions for large-amplitude swings overlap with the ones of all the molecules, the fat tale that we find for intermediate times becomes less and less pronounces and at the order of 100 ps the time series become less and less correlated. The error bars are generated by computing the standard deviation from 10 bootstrap samples of the original data.

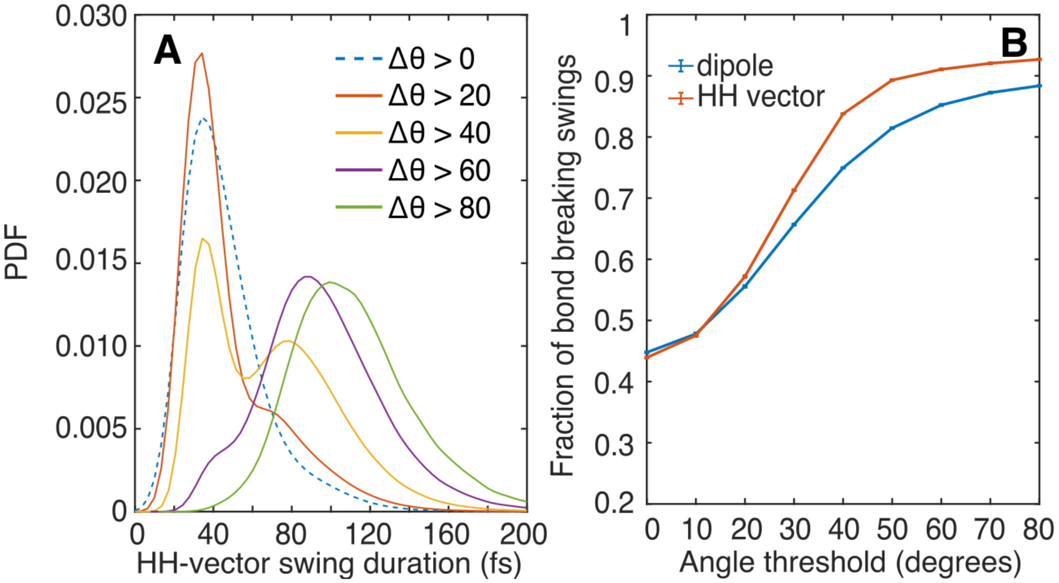

Supplementary Figure 4. Analysis of the angular swings duration, magnitude, and H-bond network local topology. (A) Probability distributions of duration Δt of swings with the angular magnitude $\Delta\Theta$ greater than a certain threshold, detected from HH vectors time series, respectively. (B) Fraction of events in which we detect H-bond breaking, depending on the angular swing amplitude. The x-axis represents a threshold angle for the HH (red) and dipole (blue) vector, so that we count all swings with an amplitude larger than the threshold value. For swings with large amplitudes, most events involve hydrogen bond breaking.

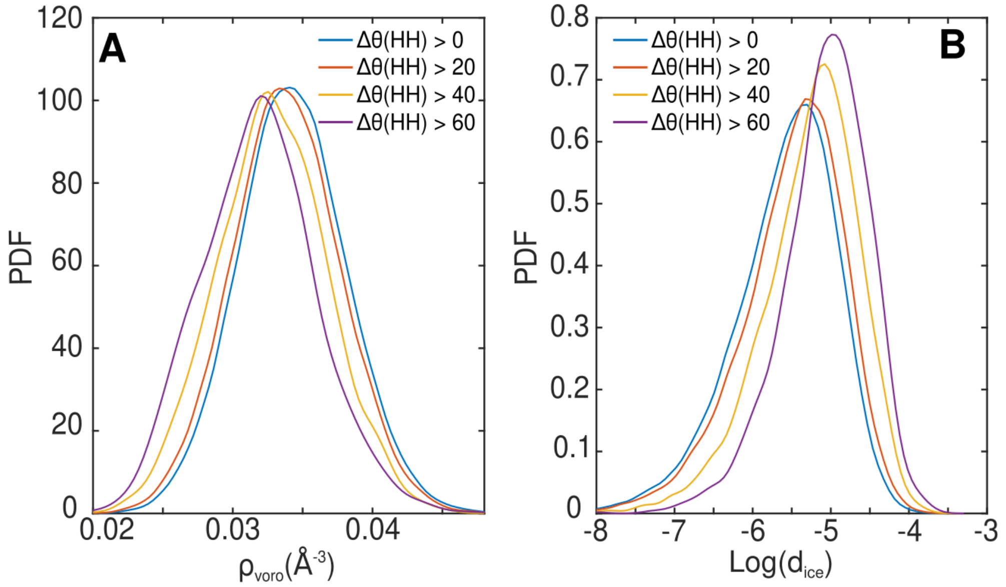

Supplementary Figure 5. (A) Probability distributions of the Voronoi density, ρ_{voro}, for the water molecules undergoing angular swings constrained by an HH-vector magnitude threshold. The probability distributions shifts towards lower local densities as we restrict ourselves to swings with the larger angular magnitude. (B) Probability distributions measuring the extent of the similarity of the local environments generated during angular jumps in liquid water, to that in hexagonal ice, namely $\log(d_{\text{ice}})$. Local environment of large angular swings with magnitude threshold $\Delta\theta > 60^\circ$ tends to be more disordered (less negative $\log(d_{\text{ice}})$) with respect to that of the small angular swings.

Supplementary Note 2

In addition to the simulations of the SPC/E water model for the small system of 1019 water molecules in a cubic periodic box (box size 31.1970 Å), we have performed simulations for larger system sizes consisting of 3400 (box size 46.720 Å) and 8152 water molecules (box size 62.5572 Å) and repeated several of our analysis. In Supplementary Fig.6, we show the distribution of the number of defects and the fraction of jumping molecules for different system size. While the average concentration of the defects as well as the average fraction of jumping waters does not depend on the box size, their relative fluctuations decrease with the increase of the system size. However, the coupling between these two quantities still persists for larger systems, as one can see from the time series associated with the number of jumps and defects for the mid-sized box of 3400 water molecules shown in Supplementary Fig.7, along with the correlation plots. Moreover, the spatial correlations between the concurrently jumping molecules are not sensitive to finite size effects, as can be seen from the distributions of distances from a jumping water to the first 4 nearest jumping molecules vs the randomly chosen ones. We compare the distributions of these distances for different system sizes for swings with magnitude larger than 60° and 40° in Supplementary Figures 8 and 9, respectively. We find that all the spatial distributions essentially overlap with each other for all three system sizes. This confirms that the collective reorientation mechanism is not sensitive to finite size effects.

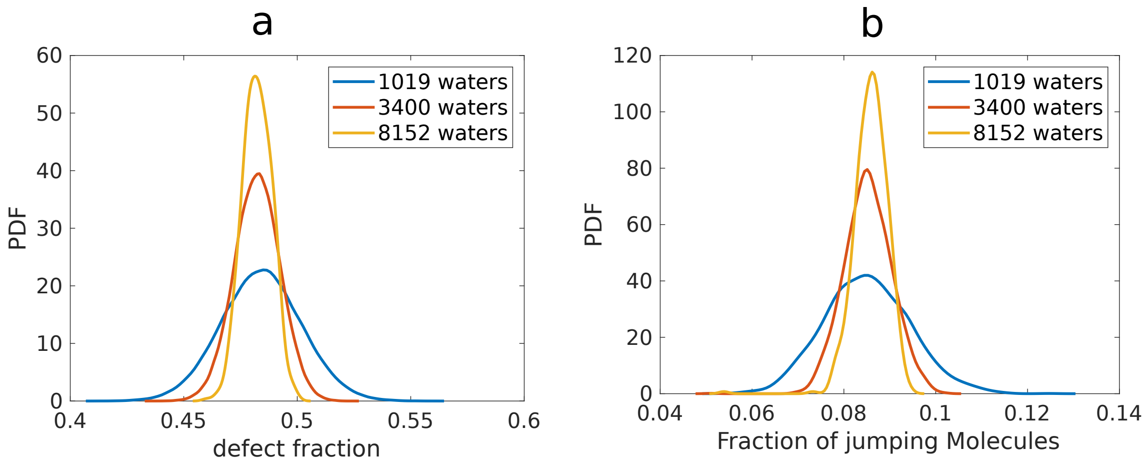

Supplementary Figure 6. (a) Distribution of the fraction of defects for the SPC/E water model for different system size. (b) Distribution of the fraction of molecules performing large angular swings ($\Delta\theta > 60^{\circ}$) within a time window of 200 fs for different system size.

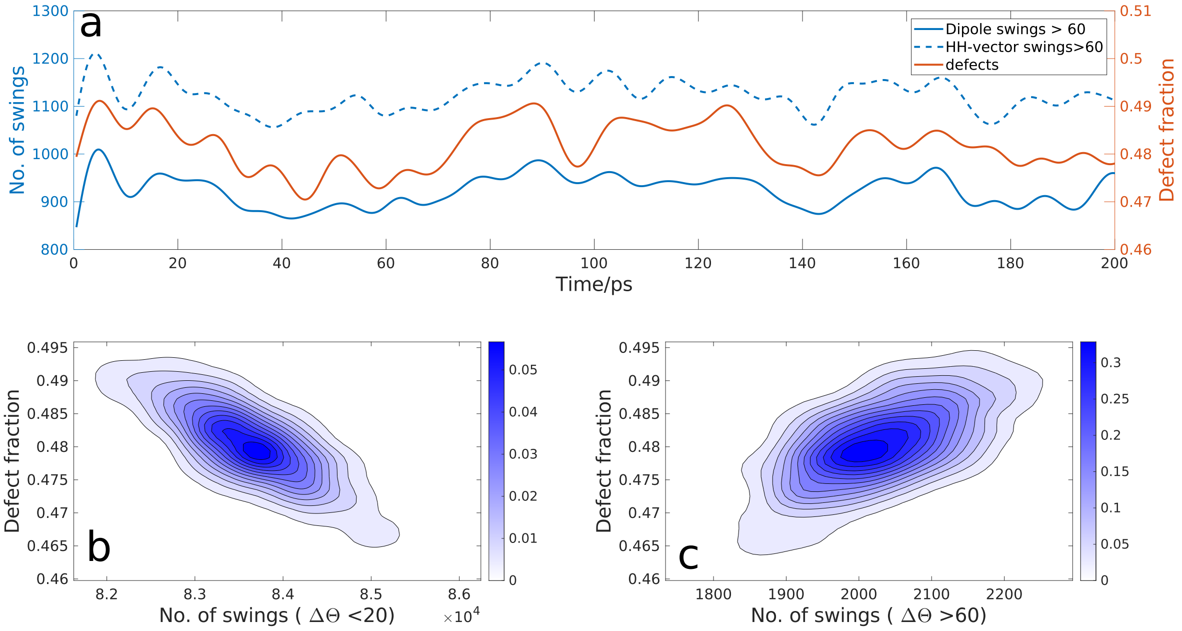

Supplementary Figure 7. Correlation between the number of simultaneous large angular swings and the number of defects in the system of 3400 molecules for the SPC/E water model, to be compared to Fig.3 in the manuscript obtained for the system of 1019 waters. The correlation coefficients obtained are (b) 0.785 and (c) 0.623.

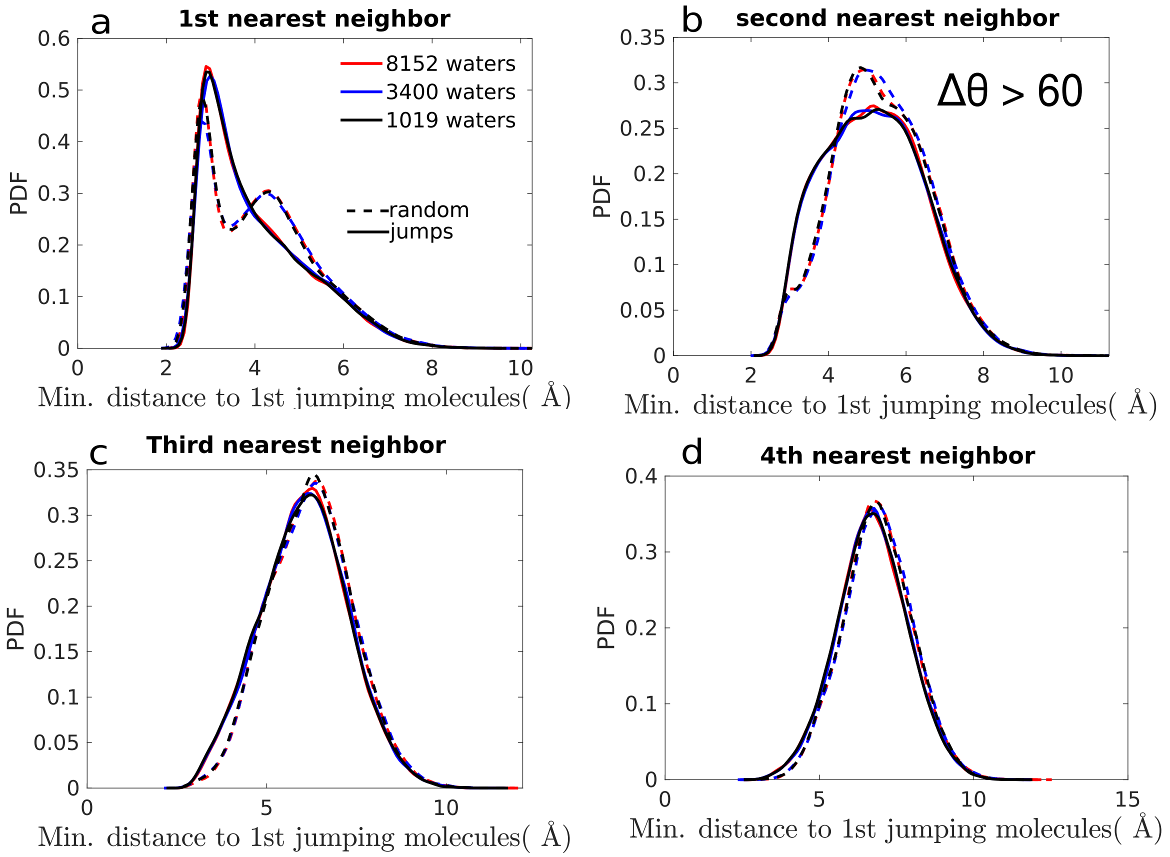

Supplementary Figure 8. Spatial correlations between concurrently jumping molecules for SPC/E water model for systems of different size inspected through the distributions of distances from a jumping water to the first 4 nearest jumping molecules (full lines) compared with those to the first 4 nearest randomly chosen molecules (dashed lines). Jumping molecules are considered to be those that perform swings larger than 60° within 200 fs time interval.

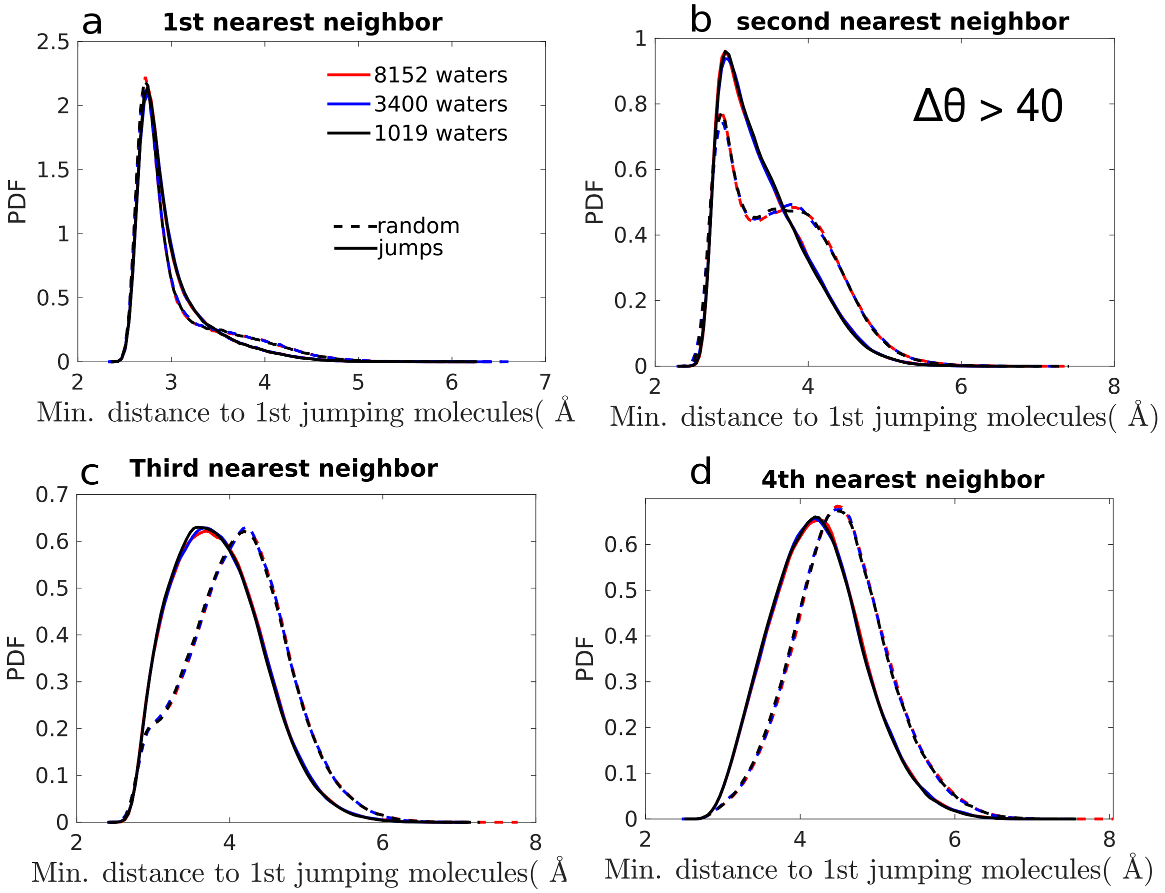

Supplementary Figure 9. Spatial correlations between concurrently jumping molecules for SPC/E water model for systems of different size inspected through the distributions of distances from a jumping water to the first 4 nearest jumping molecules (full lines) compared with those to the first 4 nearest randomly chosen molecules (dashed lines). Jumping molecules are considered to be those that perform swings larger than 40° within 200 fs time interval.

Supplementary Note 3

We examine the sensitivity of the collective reorientational mechanism to the choice of the water model. In particular, we compared the rigid SPC/E to the flexible version of SPC/E (1) and the TIP5P water model (2). Here we show detailed results of the defects and swings analysis for a box of 1019 water molecules. In particular, we analyzed the correlation between the number of defects and the number of simultaneous large jumps in the system in Supplementary Fig.10 for the TIP5/P and in Supplementary Fig.16 for the flexible SPC/E water model; spatial correlations between simultaneous large jumps with the magnitude $\Delta\theta > 60^\circ$ (Supplementary Fig.11 and Supplementary Fig.18) and with $\Delta\theta > 40^\circ$ (Supplementary Fig.12 and Supplementary Fig.19). We also compared how the distribution of the fraction of the jumping molecules and of the defect fraction in the entire system change for different water models, both for the TIP5/5 (Supplementary Fig.13) and the flexible SPC/E water model (Supplementary Fig.17) with respect to the rigid SPC/E model. Furthermore, we investigated in more details the enhancement of the collective reorientations in the TIP5/P model by looking at how different are the concentration of various defect types (see Supplementary Fig.14) and also how this affects the asymmetry of the hydrogen bonds that break on the donating and accepting side (see Supplementary Fig.15).

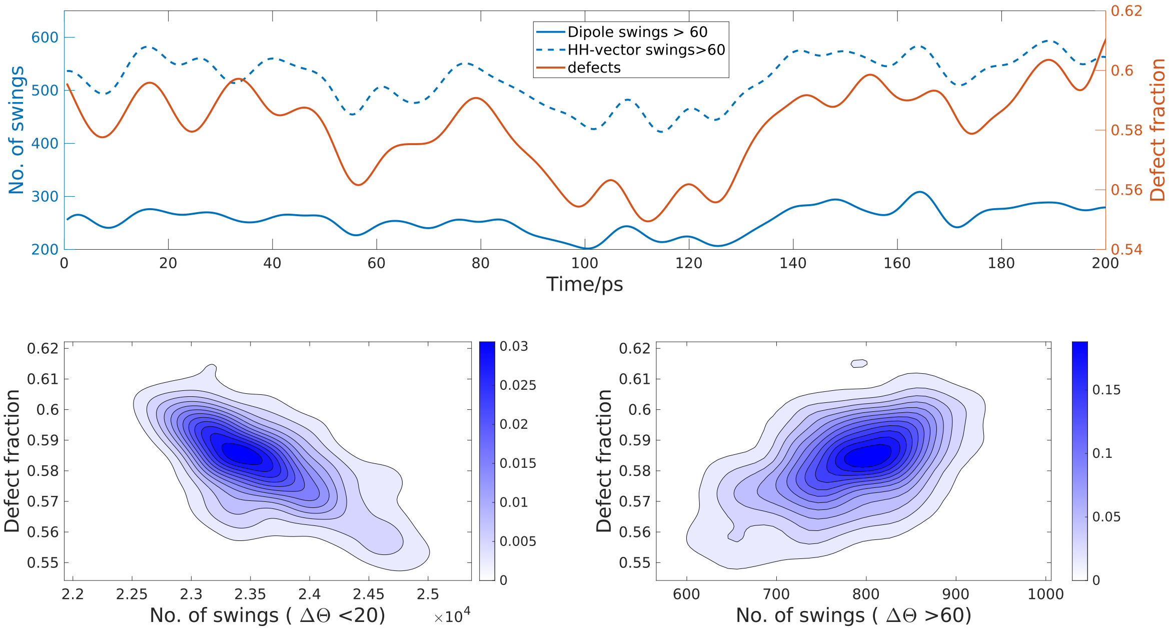

Supplementary Figure 10. Correlation between the number of simultaneous large angular swings and the number of defects in the system of 1019 water molecules for the TIP5P water model, to be compared to Fig.3 in the manuscript obtained for the SPC/E water model. The correlation coefficients obtained are (b) 0.56 and (c) 0.4596.

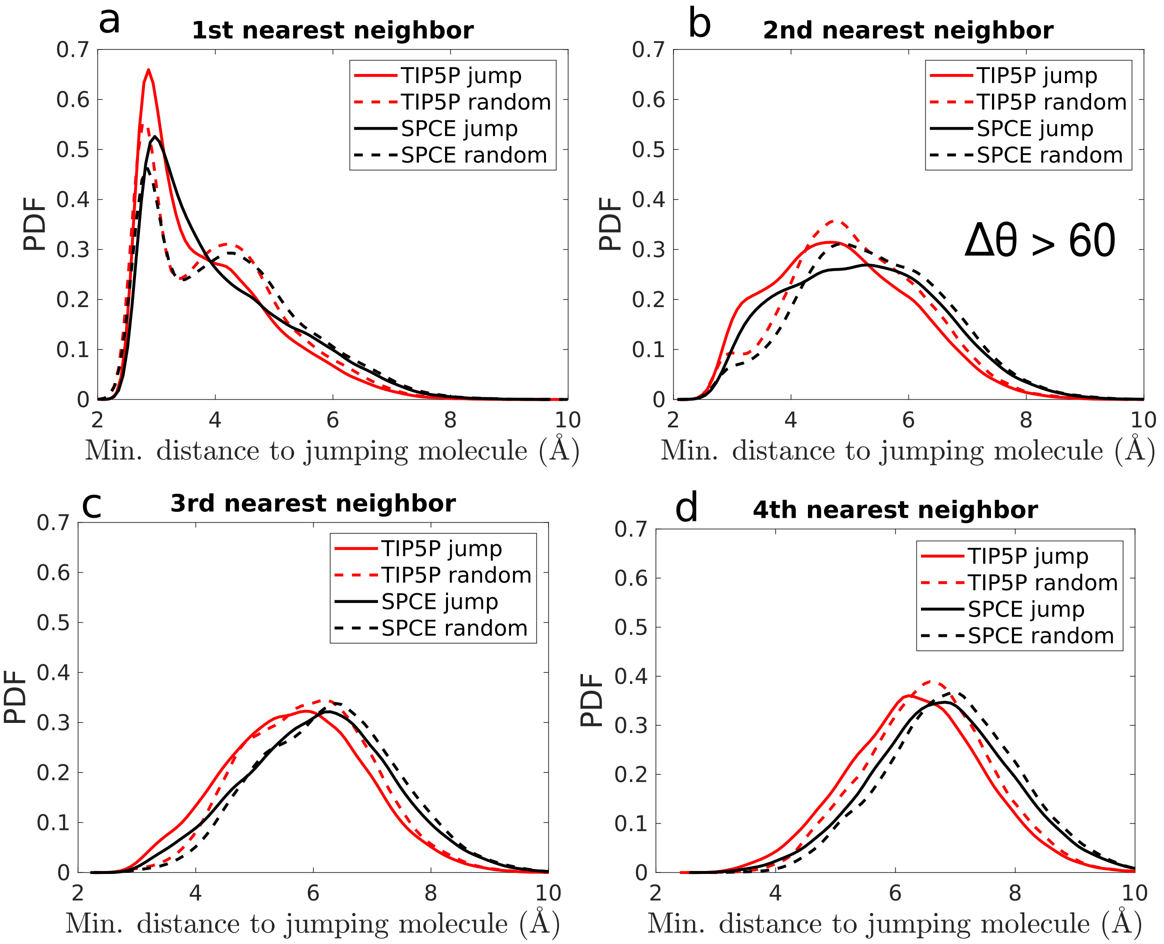

Supplementary Figure 11. Spatial correlations between concurrently jumping molecules for TIP5P water model inspected through the probability distribution functions of the distances from a jumping water to the first 4 nearest jumping molecules (full lines) compared with those to the first 4 nearest randomly chosen molecules (dashed lines). Jumping molecules are considered to be those that perform swings larger than 60° within 200 fs time interval.

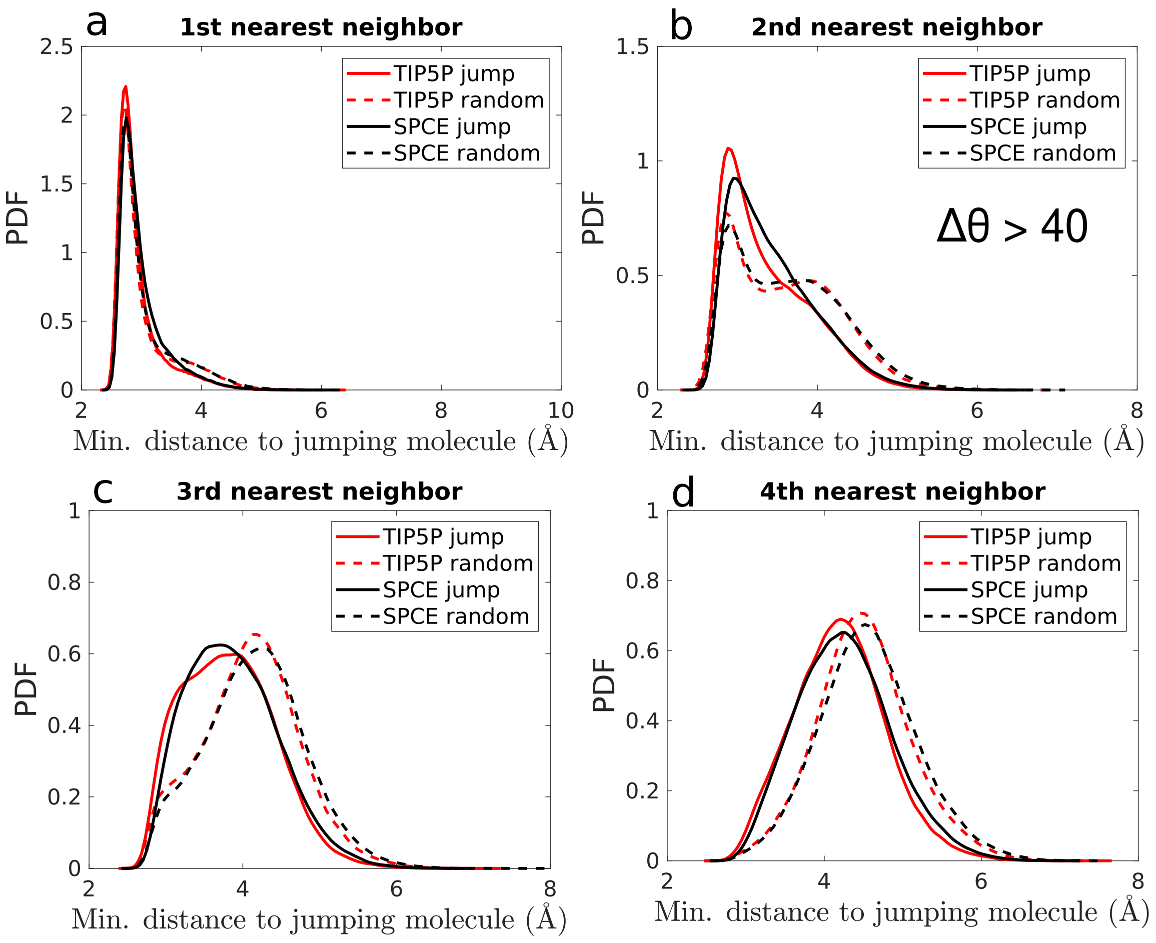

Supplementary Figure 12. Spatial correlations between concurrently jumping molecules for TIP5P water model inspected through the probability distribution functions of the distances from a jumping water to the first 4 nearest jumping molecules (full lines) compared with those to the first 4 nearest randomly chosen molecules (dashed lines). Jumping molecules are considered to be those that perform swings larger than 40° within 200 fs time interval.

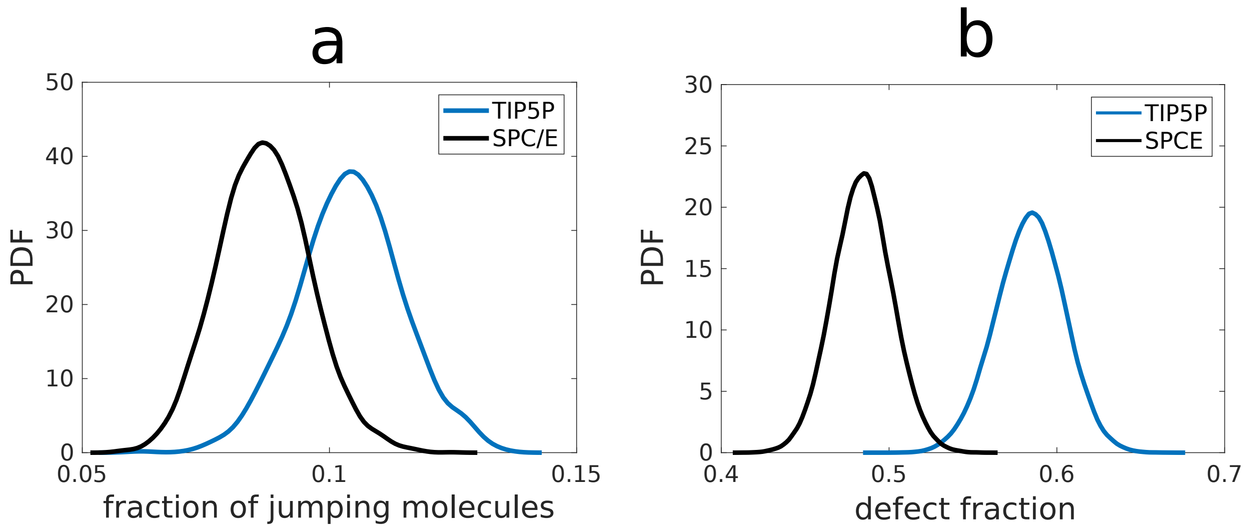

Supplementary Figure 13. Comparison for the SPC/E and TIP5P water model for a system of 1019 molecules. (a) Distribution of the fraction of jumping water molecules with $\Delta\theta > 60^{\circ}$ within 200 fs. (b) Distribution of the fraction of defects.

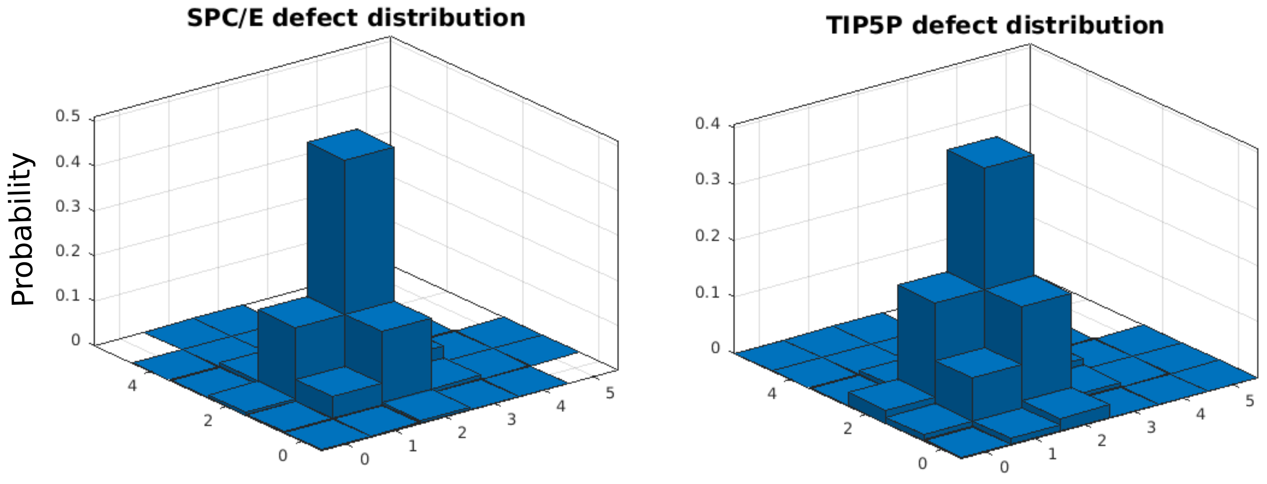

Supplementary Figure 14. Distribution of local defects for the SPC/E and TIP5P water model for a system of 1019 molecules. The axis in the plane depict number of ingoing and outgoing hydrogen bonds.

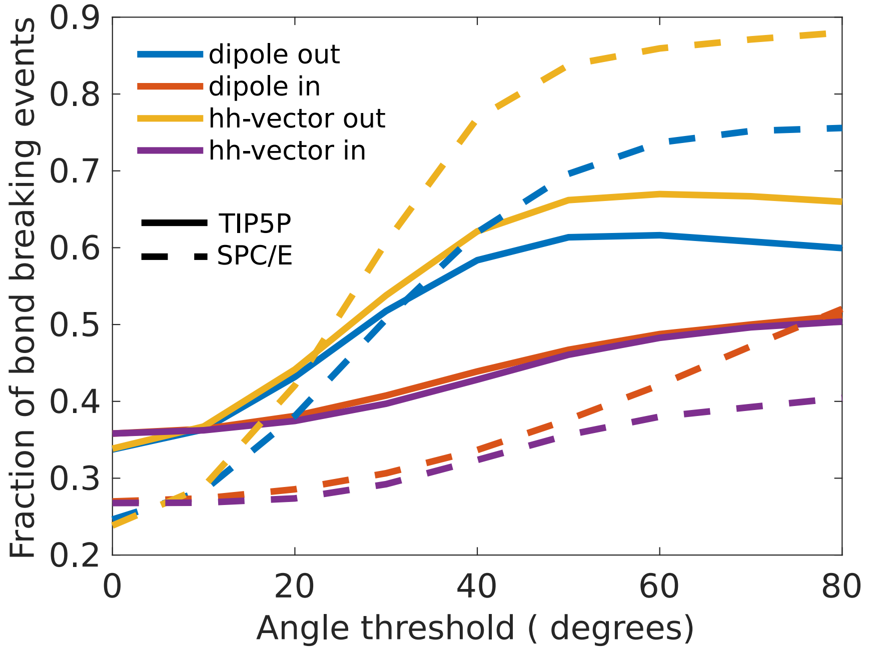

Supplementary Figure 15. Fraction of swings of magnitude greater than certain angle threshold $\Delta\Theta$ that break bonds for the TIP5P (full lines) and the SPC/E (dashed lines).

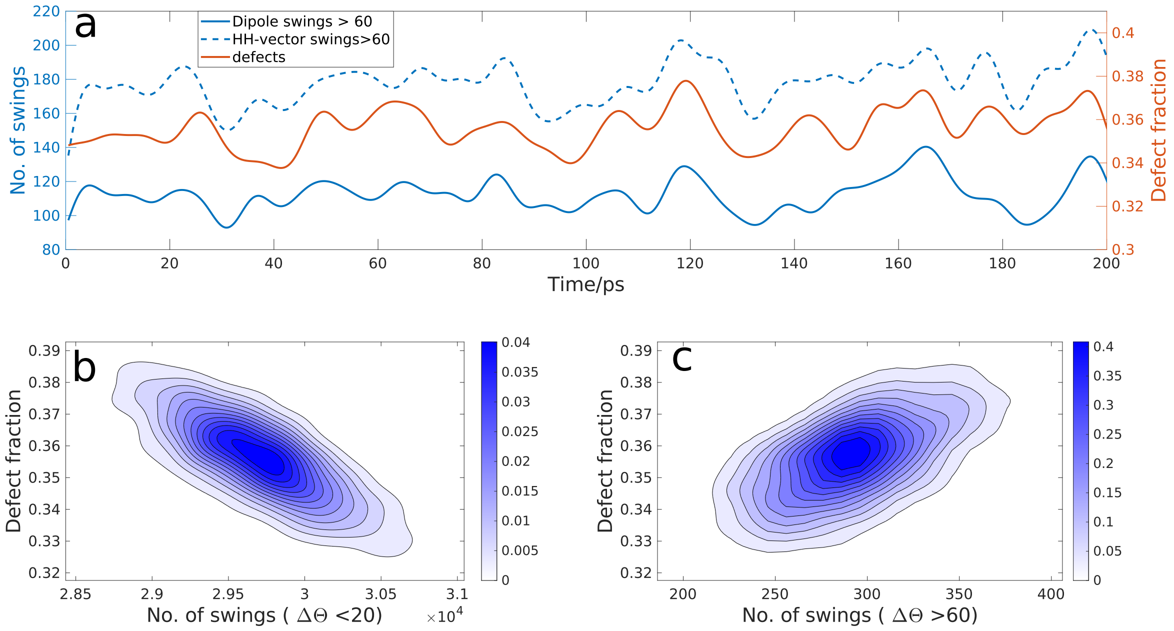

Supplementary Figure 16. Correlation between the number of simultaneous large angular swings and the number of defects in the system of 1019 water molecules for the SPC/E flexible water model, to be compared to Fig.3 in the manuscript obtained for the SPC/E rigid water model. The correlation coefficients obtained are (b) 0.61 and (c) 0.50

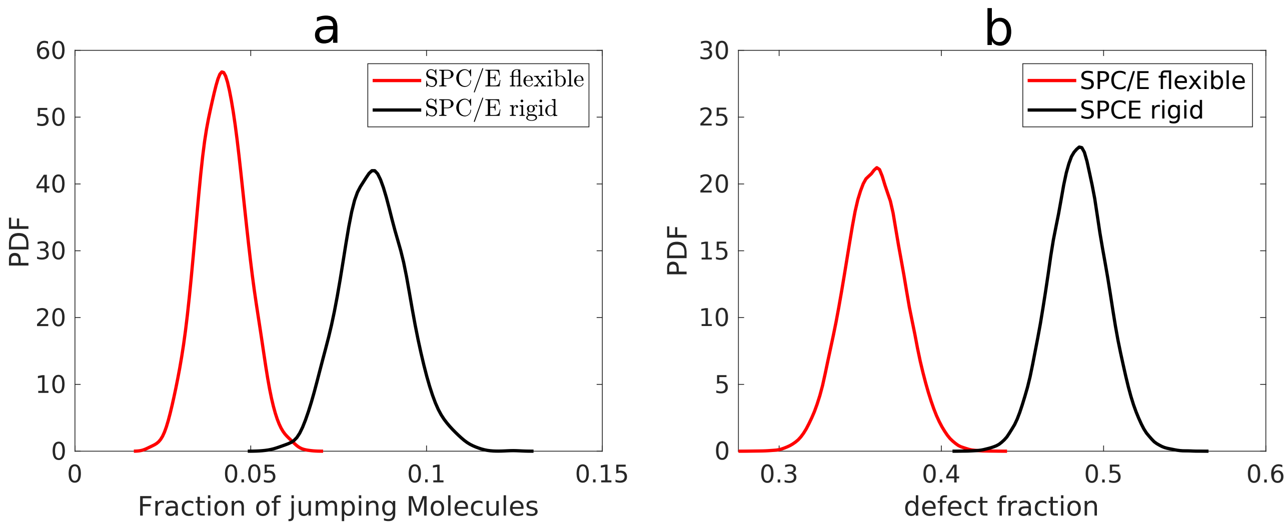

Supplementary Figure 17. Comparison for the SPC/E rigid and SPC/E flexible water model for a system of 1019 molecules. (a) Distribution of the fraction of jumping water molecules with $\Delta\theta > 60^{\circ}$ within 200 fs. (b) Distribution of the fraction of defects.

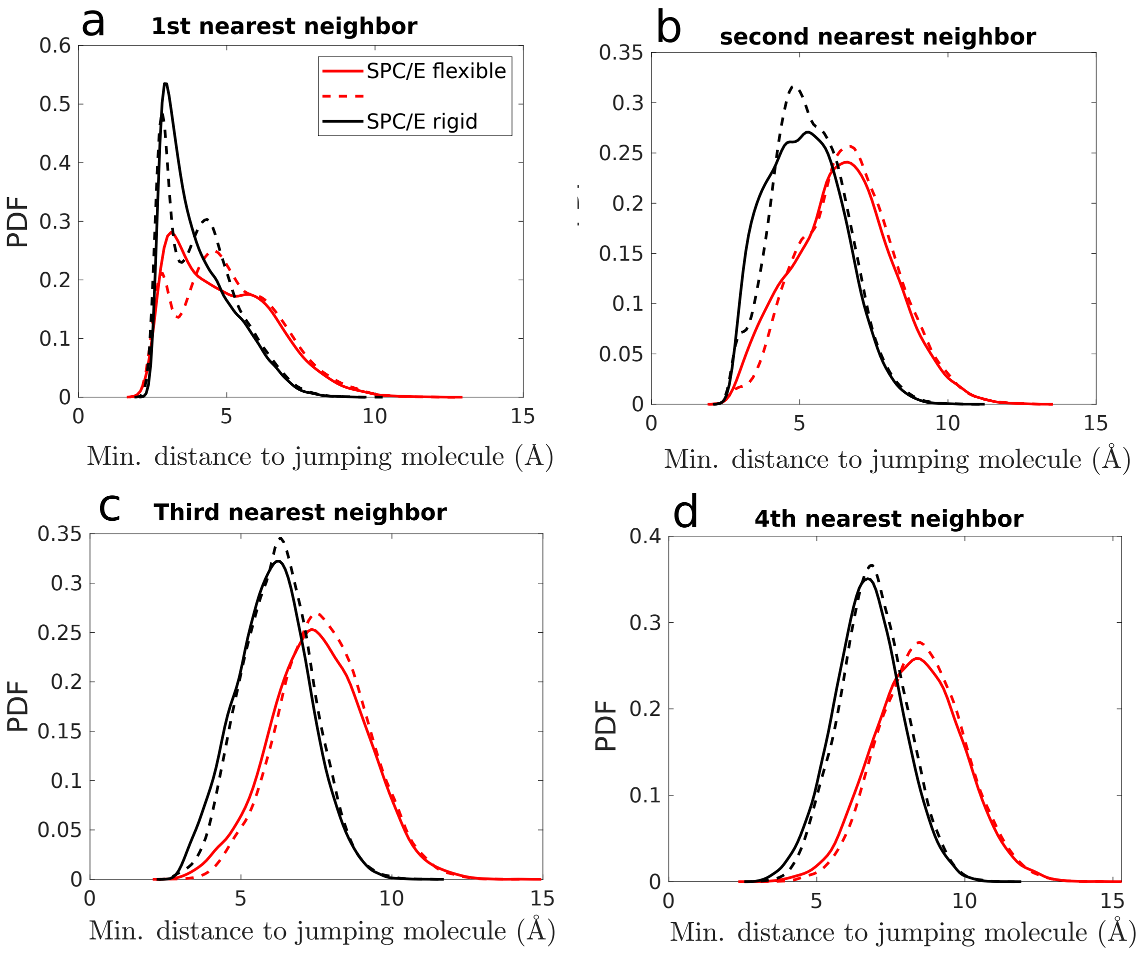

Supplementary Figure 18. Spatial correlations between concurrently jumping molecules for SPC/E flexible water model inspected through the probability distribution functions of the distances from a jumping water to the first 4 nearest jumping molecules (full lines) compared with those to the first 4 nearest randomly chosen molecules (dashed lines). Jumping molecules are considered to be those that perform swings larger than 60° within 200 fs time interval.

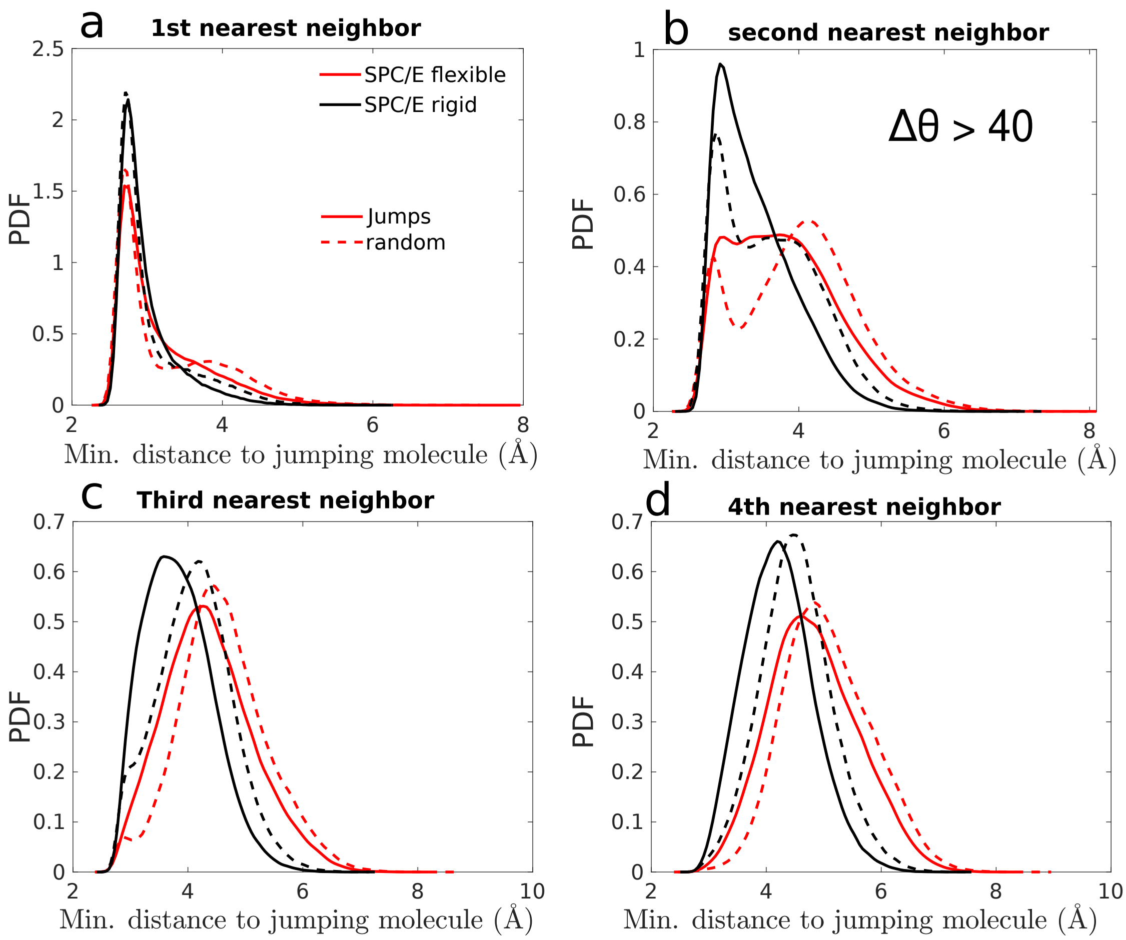

Supplementary Figure 19. Spatial correlations between concurrently jumping molecules for SPC/E flexible water model inspected through the probability distribution functions of the distances from a jumping water to the first 4 nearest jumping molecules (full lines) compared with those to the first 4 nearest randomly chosen molecules (dashed lines). Jumping molecules are considered to be those that perform swings larger than 40° within 200 fs time interval.

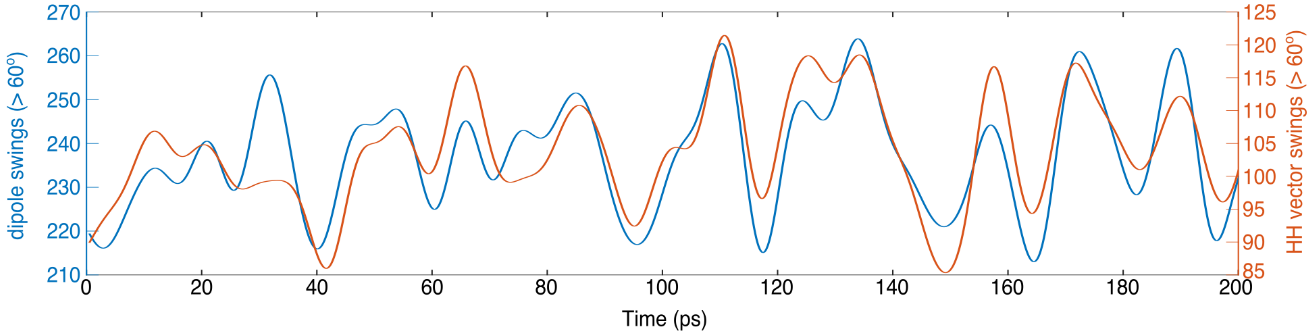

Supplementary Figure 20. Time series of MB-pol simulation of 512 water molecules at 298K(3). Time series of the number of molecules in the H-bond network performing large angular swings (amplitude larger than 60°) at each moment of time as detected from the observation of the dipole vector (blue line), or the HH vector (red line). At each moment of time, we count the number of swings happening in the system within a time window of 1ps around it.

Supplementary References

1. Yujie Wu, Harald L. Tepper, and Gregory A. Voth. Flexible simple point-charge water model with improved liquid-state properties. *The Journal of Chemical Physics*, 124(2):024503, 2006. . URL https://doi.org/10.1063/1.2136877.
2. Michael W. Mahoney and William L. Jorgensen. A five-site model for liquid water and the reproduction of the density anomaly by rigid, nonpolarizable potential functions. *The Journal of Chemical Physics*, 112(20):8910–8922, 2000. . URL https://doi.org/10.1063/1.481505.
3. Volodymyr Babin, Claude Leforestier, and Francesco Paesani. Development of a "first principles" water potential with flexible monomers: Dimer potential energy surface, vrt spectrum, and second virial coefficient. *Journal of chemical theory and computation*, 9(12):5395–5403, 2013.
